# Phosphorylation of STAT3 at Tyr705 regulates MMP-9 production in epithelial ovarian cancer

**DOI:** 10.1371/journal.pone.0183622

**Published:** 2017-08-31

**Authors:** Zan-Hui Jia, Yan Jia, Feng-Jun Guo, Jun Chen, Xi-Wen Zhang, Man-Hua Cui

**Affiliations:** Department of Obstetrics and Gynecology, The Second Hospital of Jilin University, Changchun, Jilin Province, People’s Republic of China; University of South Alabama Mitchell Cancer Institute, UNITED STATES

## Abstract

Ovarian cancer’s poor progression is closely associated with overexpression of matrix metalloproteinase 9 (MMP-9), which belongs to the class of enzymes believed to be involved in the degradation of extracellular matrix. However, the mechanisms underlying regulation of MMP-9 are not completely understood. STAT (signal transducer and activator of transcription) family of transcription factors is well known to be engaged in diverse cellular functions. Activation of STAT3 has been observed in a number of cancers, promoting tumorigenesis and metastasis via transcriptional activation of its target genes. In this study, we tested our hypothesis that STAT3 regulates *MMP-9* gene expression in epithelial ovarian cancer. Using epithelial ovarian cancer cell lines as *in vitro* model, we show an abundance of phosphorylated STAT3 at Tyr705 (p-STAT3) in SKOV3 cell line. We further show that *MMP-9* gene promoter was significantly enriched by p-STAT3, and IL-6 treatment led to a significant increase of MMP-9 at mRNA and protein levels, in addition to an association of p-STAT3 with *MMP-9* gene. By using luciferase reporter assay, we determined that the STAT3 DNA responsive element of MMP-9 was sufficient to regulate transcriptional activity of a heterologous promoter. These results suggest that the phosphorylation of STAT3 regulates MMP-9 production in ovarian cancer, which might be responsible for its invasiveness and metastasis.

## Introduction

Epithelial ovarian cancer (EOC) is one of the leading causes of cancer-related deaths in industrialized countries [1]. Despite advances in early diagnosis and treatment, the 5-year survival rate of ovarian cancer patients has not significantly improved. In the United States, a significantly high number of patients die of ovarian cancer each year [2]. This is simply due to lack of complete understanding of the etiological factors underlying ovarian carcinogenesis, local invasiveness and distant metastasis. The most commonly administered chemotherapy for the treatment of ovarian cancer is a combination of carboplatin and paclitaxel [3, 4]. Up to 80% of the patients initially respond well to this therapy [5, 6], however, a majority of patients suffer from recurrence and often die due to local and distant metastasis. Obviously, identification of novel drug targets and subsequent development of novel therapeutic approaches is needed.

In normal physiological processes, matrix metalloproteinase (MMP) family is closely associated with the degradation of extracellular matrix. This includes tissue remodeling as well as numerous pathological processes, which are exemplified by arthritis and malignant metastasis [7–11]. MMPs and vascular endothelial growth factor have been reported to promote tumor invasion and angiogenesis. MMP-9, a widely investigated MMP, is actively engaged in pathological remodeling processes that are comprised of inflammation and metastasis. In addition, MMP-9 directly breaks down extracellular matrix (ECM) proteins and increases cytokines and chemokines for regulation of tissue remodeling. MMP-9 depletion has been proven overall beneficial in multiple animal models of cancer diseases [10]. To this end, molecular characterization of MMP-9 regulation is critical for development of novel therapeutic approaches for treatment of ovarian cancer.

STAT family belongs to intracellular transcription factors that mediate many physiological and pathological processes, including cell proliferation, differentiation and apoptosis. Activation of STAT3, a prominent member of STAT family, is implicated in cell differentiation, development, proliferation, inflammation and apoptosis [12–14]. Enhanced activity of STAT3 has been observed in a wide variety of human tumors, as a means for the cancer cells to escape therapeutic insult, and survive. These include hematologic as well as solid malignancies [15]. Cytokines and growth factors lead to phosphorylation of STAT3 by receptor-associated Janus kinases, leading to formation of homo- or heterodimers, and translocation of STAT3 to the nucleus where it functions as a transcription activator [16]. In response to ligands (interferons, epidermal growth factor and interleukins), STAT3 is activated by phosphorylation at tyrosine-705 (Tyr705).

In the present study, we tested whether STAT3, phosphorylated at Tyr705, functions as a transcriptional activator, for positive regulation of MMP-9 in epithelial ovarian cancer. We show that depletion of STAT3 leads to down-regulation of MMP-9, but IL-6 treatment results in an increased phosphorylated STAT3 at Tyr705 (p-STAT3) and MMP-9, at mRNA and protein levels. Our results support that p-STAT3 may represent a potential therapeutic target in epithelial ovarian cancer.

## Materials and methods

### Epithelial ovarian cancer cell lines

The epithelial ovarian cancer cell lines SKOV3[17], RMG-1, MCAS, and RMUG-1 [18], and HEK293T cells were obtained from ATCC (Manassas, VA), and cultured in RPMI-1640 media containing 10% FBS at 37°C and 5% CO_2_ prior to further analysis.

### Antibodies and reagents

Antibodies for Western blot included anti-STAT3 (Millipore), p-STAT3 Tyr705 (Cell Signaling), anti-MMP-9 (Santa Cruz Biotech, TX), and anti-GAPDH (Sigma-Aldrich, MO). Recombinant human interleukin-6 (IL-6) was purchased from Sigma-Aldrich.

### Gene silencing

Cells were plated in 6-well culture plates in 3 ml of RMPI-1640 media containing 10% FBS, and transfected for 48 hours, using FlexTube siRNA (Qiagen, Valencia, CA) targeting STAT3-Lipofectamine 2000 (3μl; Invitrogen), as per manufacturer’s instructions with a final concentration of siRNA at 50 mM. A non-targeting siRNA was used as a negative control.

### Reverse transcription PCR (RT-PCR)

We extracted total RNAs using Trizol (Invitrogen, CA). Total cDNA was synthesized using oligo-dT/random primers and Superscript II Reverse Transcription kit (Invitrogen) for detection of target mRNAs. Quantitative PCR was performed on ABI StepOne qPCR System (ABI, USA). The following primers (forward/reverse) were used: MMP-9: 5’- gggaagatgctgctgttca-3’/5’-tcaactcactccgggaactc-3’, STAT3: 5’-ggtgagacttgggcttacca-3’/5’- ctttaatgggccacaacagg-3’; and GAPDH: 5’-cagtggtggacctgacct-3’/5’-ctgtagccaaattcgttg-3’.

### Immunofluorescent staining (IF)

4% paraformaldehyde (in PBS) was used to fix cells. This was followed by permeabilization in 0.5% Triton X-100 (in TBS) for 10 minutes at room temperature, washing with TBS and incubation with p-STAT3 antibody at room temperature for 1 hr. Cells were washed once again with TBS (containing 0.1% BSA) and then exposed to secondary antibodies (Alexa Fluor 488 goat anti-rabbit IgG) for 1 hr at room temperature. Finally, cells were stained with DAPI (Invitrogen) and visualized.

### Chromatin immunoprecipitation (ChIP)

ChIP was conducted as previously reported [19]. Briefly, cells were cross-linked (using 1% formaldehyde) and lysed (in ChIP lysis buffer: HEPES-KCl 50 mM, pH 7.5; NaCl 140mM; EDTA 1mM; Triton X-100 1%; sodium deoxycholate 0.1% and SDS 0.1%). They were then sonicated for the shearing of chromatin into smaller (200-500-bp) fragments. The generated fragments were then immunoprecipitated with either control rabbit IgG antibody (Cell Signaling) or ChIP-grade antibody (anti-STAT3; Millipore), and incubated with protein G agarose beads (Roche Applied Science). This was followed by reversal of cross-linking by Proteinase K treatment, and recovery of DNA. SYBR green dye (QIAGEN, Valencia, CA) was used to quantitate PCR products. Primers used for *MMP-9* gene promoter were: 5’-tggggaggatatctgacctg-3’/5’-ttgcaaactgcagagcttgt-3’.

### DNA constructs

Two complementary DNA strands containing 3 copies of STAT3 putative target site at *MMP-9* gene promoter were synthesized from IDT DNA. These two strands at equal molar concentrations were denatured for 5 min at 95°C and annealed at room temperature for 5 min. Then the DNA fragments were cloned into luciferase reporter vectors (Promega, USA) immediately upstream of TATA box of the firefly luciferase gene. Plasmid DNAs were validated by DNA sequencing.

### Dual luciferase assay

Dual luciferase assays were performed using Victor 3V (PerkinElmer, MA), following the instructions from the vendor (Promega, WI).

### Statistical analysis

The data were presented in Mean ± S.D. (N = 3). We used unpaired Student *t* test to compare the test group with the control. p<0.05 was considered statistically significant.

## Results

### STAT3 regulates MMP-9 in epithelial ovarian cancer cell

**Fig 1:**
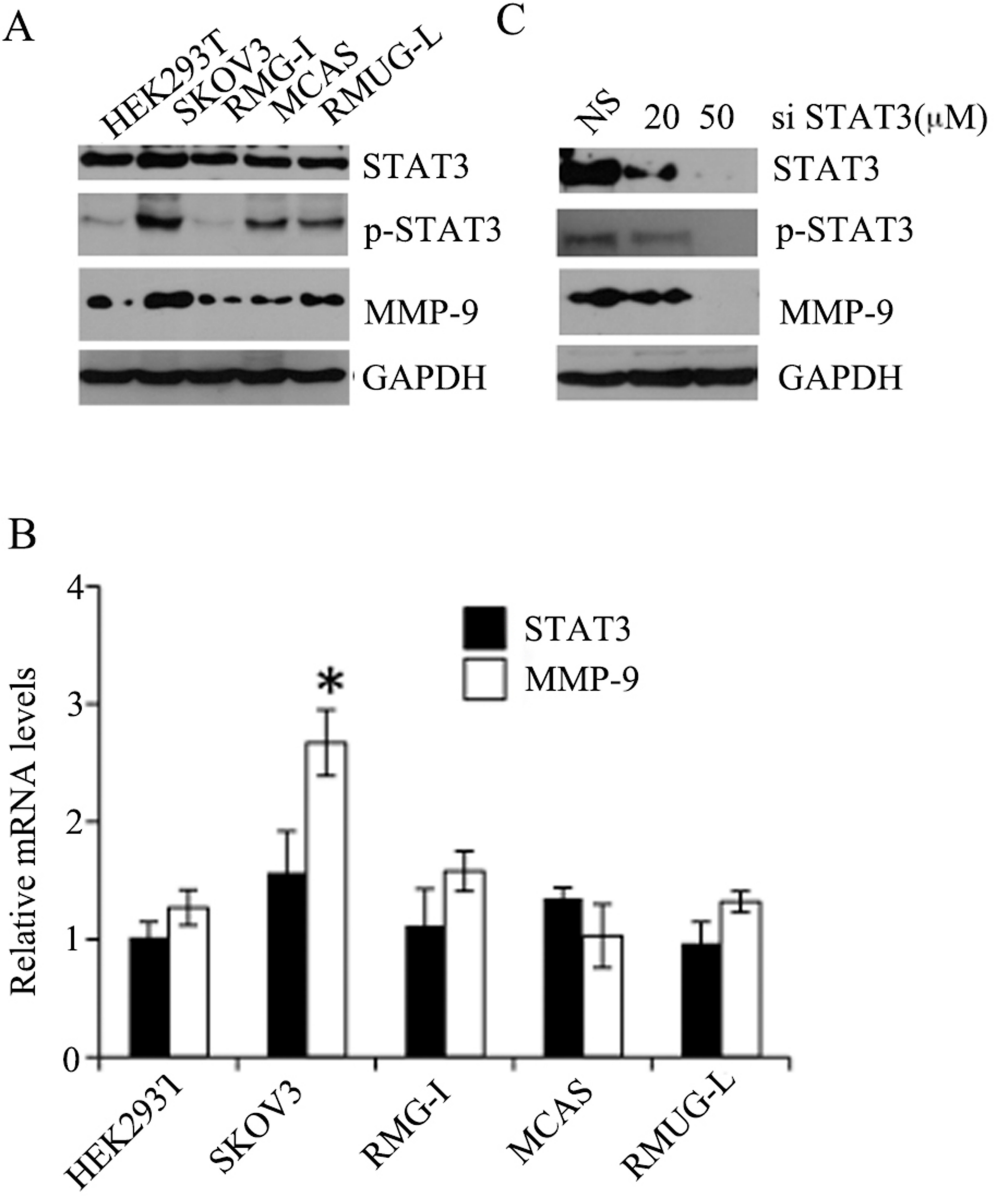
Expression of STAT3, p-STAT3, MMP-9 in ovarian cancer lines. (**A**) Total cell lysates were extracted and subjected to immune-blot analysis. This gel pattern is representative of 3 independent experiments. (**B**) Total RNAs were extracted, and RT-PCR performed for the determination of relative mRNA levels of MMP-9 and STAT3. * p< 0.01. (**C**) SKOV3 cells were transfected with siRNAs targeting STAT3 mRNA (siSTAT3) and then Western blot was performed. This gel pattern represents from 3 independent experiments. NS, non-specific siRNAs.

It has been previously reported that STAT3 was phosphorylated at Tyr705 in epithelial ovarian cancer cells [20]. In this study, we first determined the abundance of STAT3, p-STAT3, and MMP-9 in ovarian cancer cells. 4 ovarian cancer cell lines were cultured for the analysis. We used Western blot and RT-PCR to assess protein and mRNA levels, respectively. As shown in Fig 1A, while there was no obvious difference in total STAT3 protein and mRNA levels observed among these cell lines, p-STAT3 and MMP-9 were significantly increased in SKOV3 cell line, and there was a positive correlation observed between the abundance of p-STAT3 and MMP-9 (**Fig 1A and 1B**). Moreover, siRNA-mediated depletion of STAT3 caused a significant decrease of MMP-9 in SKOV3 cell line (**Fig 1C**). These results suggest that STAT3 is constitutively phosphorylated/activated in SKOV3 cell line, *MMP-9* gene is likely a target of p-STAT3, and SKOV3 cell line is a suitable model to study relation of p-STAT3 and *MMP-9* gene expression.

### Positive correlation of nuclear p-STAT3 and MMP-9 expression in SKOV3 cell

**Fig 2:**
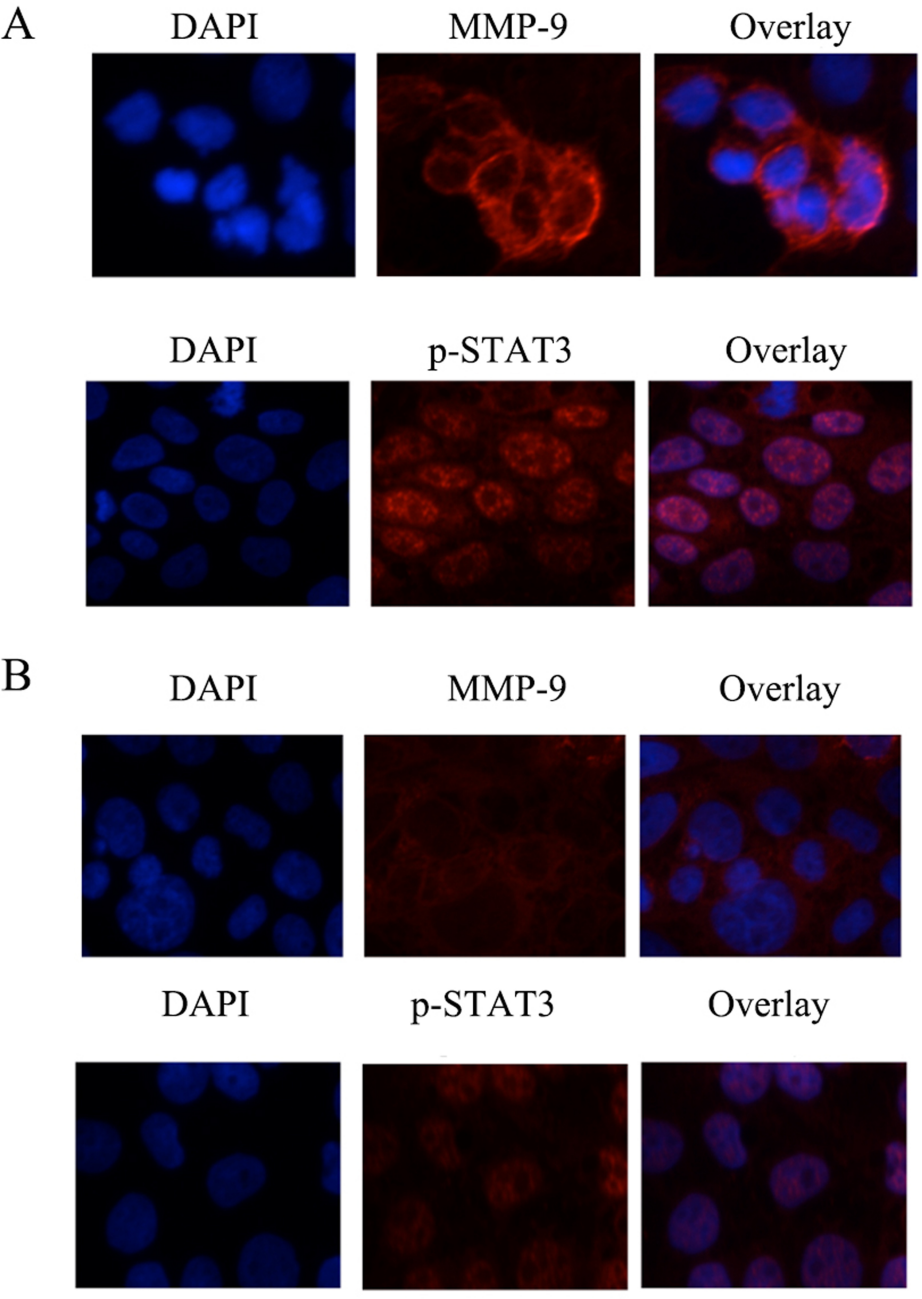
Intra-cellular distribution of MMP-9 and p-STAT3. Immunofluorescence staining (N = 3) was performed in order to directly visualize the cellular distribution pattern of p-STAT3 and MMP-9 in SKOV3 cells (**A**) or HEK293T cells (**B**). Original magnification, 200Χ.

STAT3 is a cytoplasmic transcription factor which is activated by phosphorylation, in response to various stimuli. Once activated, it translocates to nucleus, leading to regulation of multiple target genes [13]. To study the correlation between nuclear p-STAT3 and MMP-9 expression level, we used IF staining to study cellular distribution of p-STAT3. As shown in **Fig 2**, p-STAT3’s positive staining was predominantly observed in the nucleus of SKOV3 cells. However, in contrast, HEK293T cells rarely exhibited any nuclear staining of p-STAT3. In consistent with this observation, the protein level of MMP-9 was remarkably increased in SKOV3 cells, but barely detected in HEK293T cells. These findings further support that constitutively active STAT3 in SKOV3 cells might play a role in regulation of *MMP-9* gene.

### MMP-9 constitutes a direct target of p-STAT3 in SKOV3 cell

**Fig 3:**
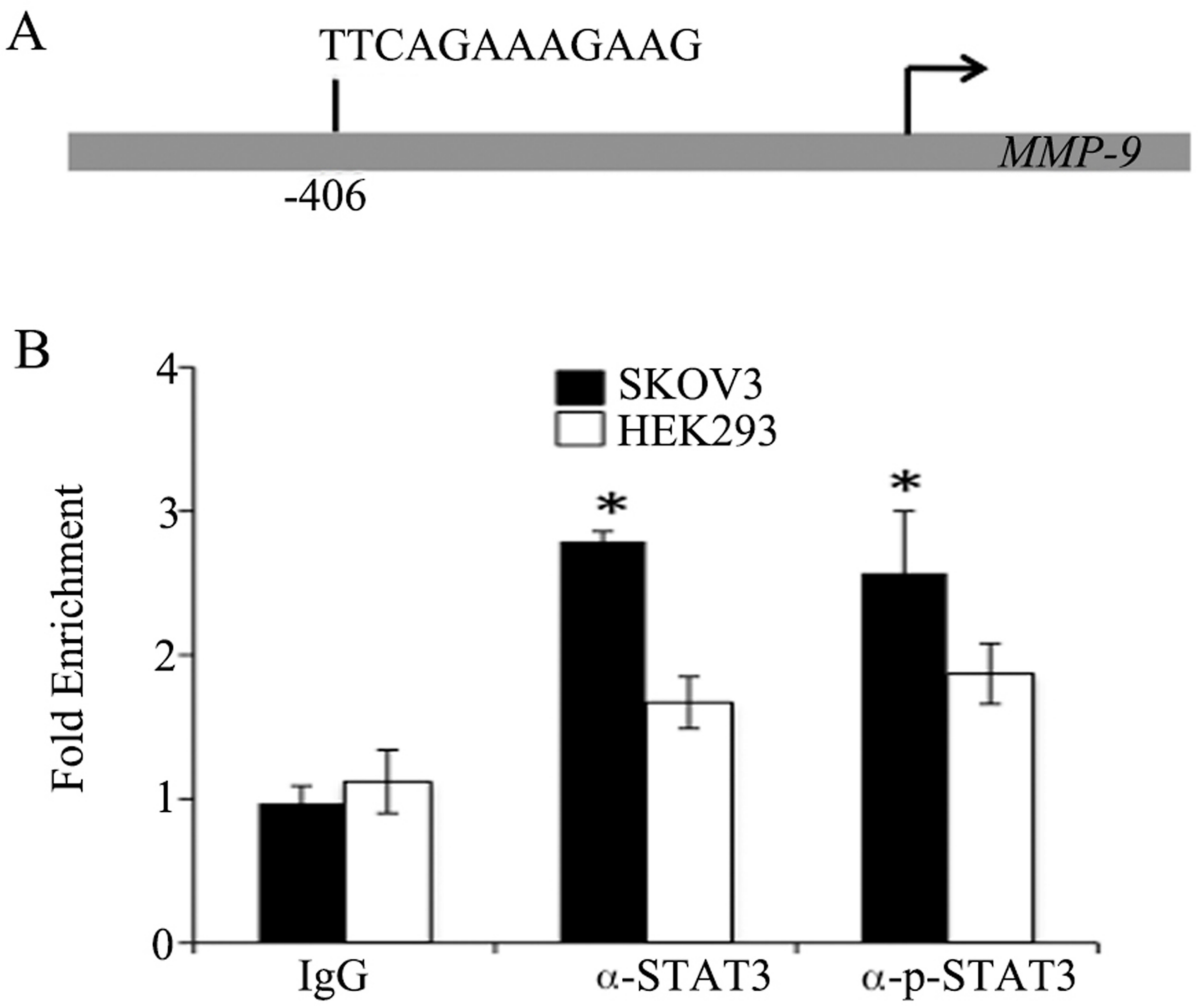
Association of p-STAT3 and *MMP-9* gene promoters. (**A**) Schematic presentation of the putative GAS sequence at *MMP-9* gene promoter. (**B**) ChIP assay was carried out to determine the occupancy of p-STAT3 at *MMP-9* gene promoter. Fold enrichment p-STAT3 was derived with normalization to a human α satellite. Rabbit IgG served as the non-specific control. The bars are indicative of the average fold *MMP-9* enrichment (Y-axis), and error bars represent standard deviation, as calculated from 3 independent experiments. * p < 0.01.

To experimentally validate that p-STAT3 directly targets MMP-9 gene, we first performed bioinformatic analysis based on p-STAT3 DNA responsive elements, also known as gamma-activated sequence (GAS) (5’-TTCNNNGAA-3’) [21], and found a DNA sequence (5’-TTCAGAAAGAAG-3’) at -406 of *MMP-9* gene promoter (**Fig 3A**). Next, we used ChIP to analyze the interaction between p-STAT3 and MMP-9 gene with the use of PCR primers encompassing this putative GAS sequence. As shown in **Fig 3B**, *MMP-9* gene promoter was significantly enriched with STAT3 and p-STAT3, compared to that by normal rabbit IgG as the basal control. Thus, we observed a positive regulation of MMP-9 by recruitment of p-STAT3 in SKOV3 cells.

### IL-6 stimulates MMP-9 production in SKOV3 cell

**Fig 4:**
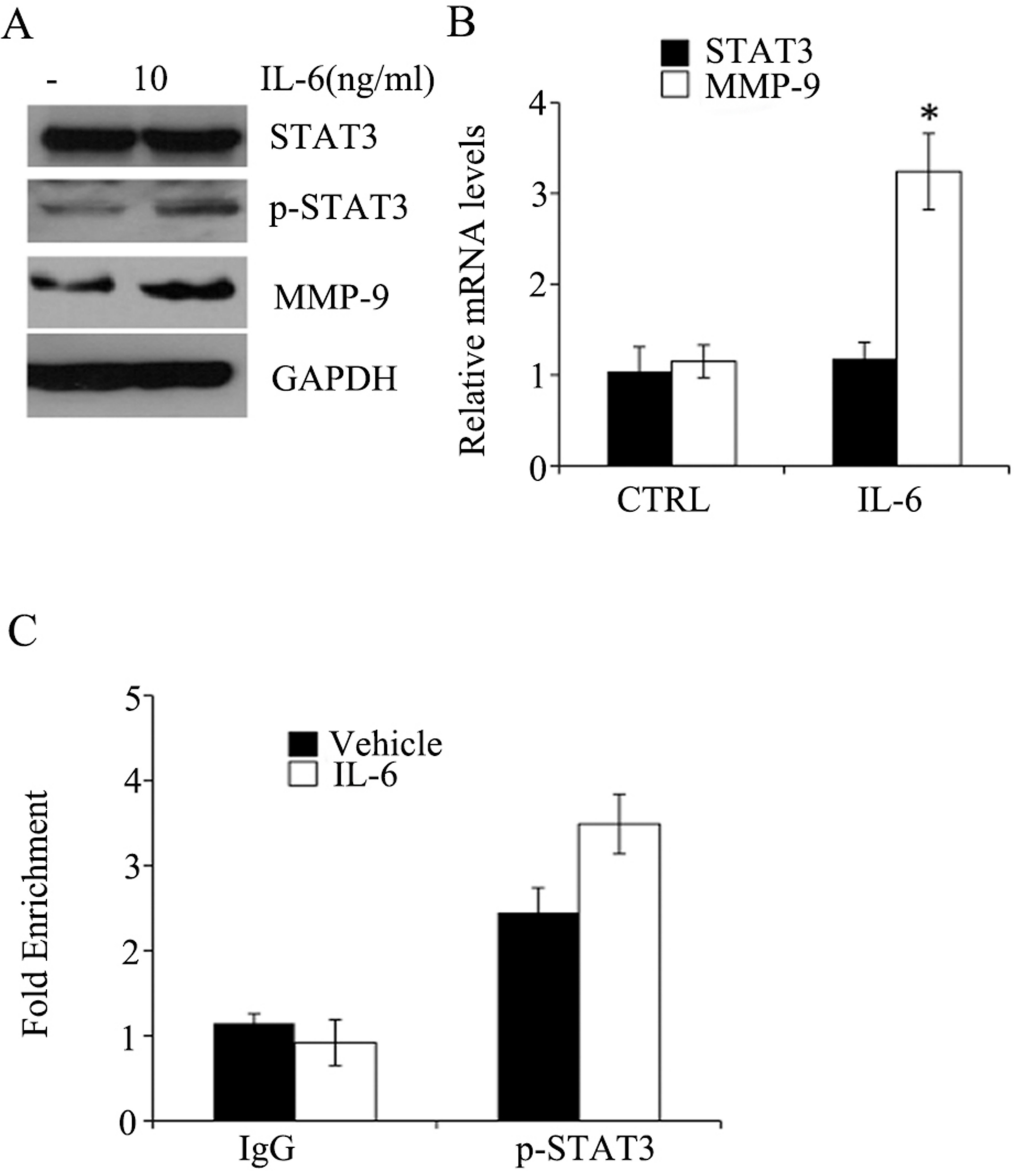
IL-6 stimulated the expression of p-STAT3 and MMP-9 in SKOV3 cells. (**A**) Western blot analysis of lysates of cells treated with or without IL-6 (N = 3). (**B**) RT-PCR was performed to determine mRNA level changes in response to IL-6 exposure (N = 3); * p<0.01. (**C**) ChIP analyses were conducted for the determination of the occupancy of p-STAT3 at *MMP-9* gene promoter in response to IL-6 treatment (N = 3).

IL-6 is a factor known to be involved in the initiation and progression of ovarian cancer [22, 23]. It acts through activation of several signaling pathways, particularly, STAT3 pathway. In order to assess the effects of IL-6 on phosphorylation of STAT3 at Tyr705, we incubated SKOV3 cells with IL-6 for 24 hrs and then conducted Western blot, RT-PCT, and ChIP to determine changes of STAT3, p-STAT3 abundances and their association with *MMP-9* gene promoter, respectively. As shown in **Fig 4**, IL-6 exposure led to accumulation of p-STAT3. In addition, IL-6 treatment had no effects on STAT3 mRNA levels, but increased *MMP-9* gene expression. Moreover, IL-6 treatment resulted in a significant increase in interaction between p-STAT3 and MMP-9 gene promoter.

Taken together, we show that IL-6 exposure stimulates MMP-9 production via promoting phosphorylation of STAT3 and its subsequent association with *MMP-9* gene.

### GAS of MMP-9 regulate transcriptional activity of a heterologous promoter

**Fig 5:**
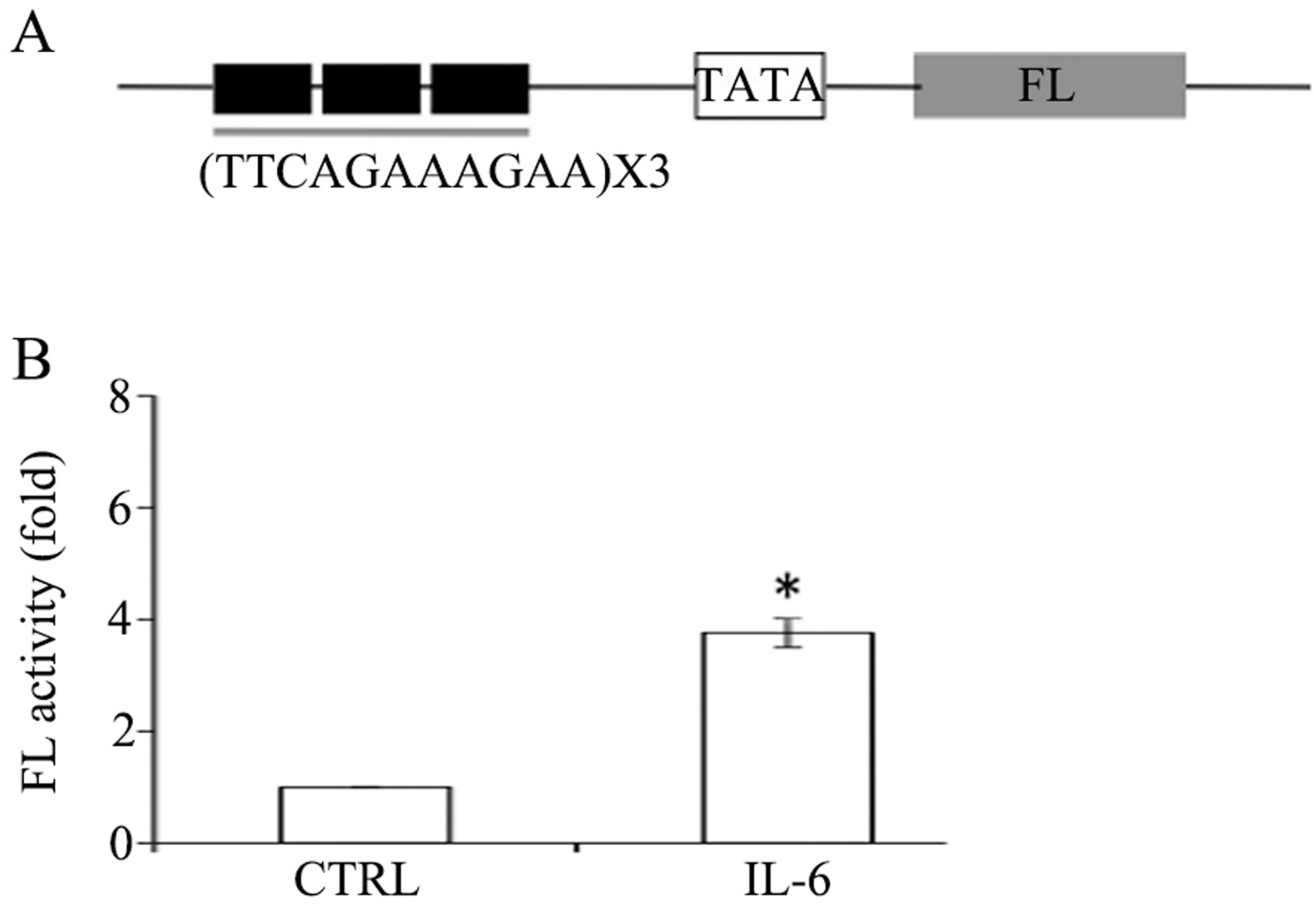
The putative GAS of *MMP-9* regulates transcription of a heterologous promoter. (**A**). Schematic presentation of luciferase reporter constructs. pGL4.32 (Promega) harbors 3 copies of GAS and a minimal promoter (TATA), which regulate expression of firefly luciferase gene (FL). pCMV-RL (*renilla luciefease*) was used as the internal control (not shown). (**B**). SKOV3 cells were co-transfected with pCMV-RL (100 ng) and the construct (1 μg) for 48 hrs and subsequently exposed to IL-6 for additional 24 hrs. Dual luciferase was performed as detailed in Material and Methods. The bars are representative of average FL fold change, with normalization to renilla luciferase activity and error bars indicate the standard deviation calculated from three independent experiments.

Three copies of GAS elements were placed upstream of a minimal promoter as previously described, to make firefly luciferase reporter (FL) constructs. As shown in **Fig 5**, IL-6 was able to stimulate FL activity by more than 3 fold. Therefore, we show that the GAS of MMP-9 is sufficient to regulate transcription to a heterologous promoter.

## Discussion

MMP-9 selectively degrades extracellular matrix (ECM), releasing several factors (such as, growth factors and cytokines) that are present in the ECM. It also promotes tumor invasiveness and metastasis by cleaving cell surface proteins adhesion molecules [24]. To this end, targeting the signaling pathway regulating MMP-9 would make an effective therapeutic approach for treatment of ovarian cancer. Activation of STAT3 signaling is known to be important for the oncogenic properties of cancer cells, including ovarian cancer cells. Effective inhibition of this signaling pathway, through the use of STAT3 inhibitors, has been observed to inhibit growth and proliferation of ovarian cancer cells. Here, we demonstrate, for the very first time, constitutive activation of STAT3 in ovarian cancer cells, and that *MMP-9* gene promoter contains a STAT3 DNA responsive element. We further show that MMP-9 is positively regulated by this constitutively activated STAT3, specifically, phosphorylation of STAT3 at Tyr705. These results clearly show that STAT3 is an attractive drug target in therapy of ovarian cancer.

IL-6 plays important roles in a variety of biologic processes in different types of cells, including the cancer cells [25–27]. Overexpression of IL-6 has been observed in many cancers, such as endometrial, lung, colorectal, breast and ovarian cancers [28, 29]. IL-6 exerts its role through activation of various downstream signaling pathways, STAT3 pathway being one of them. IL-6-STAT3 signaling axis has been shown to be relevant to pathogenesis of ovarian cancer, with phosphorylation/activation and subsequent nuclear translocation of STAT3 being frequent events in ovarian cancers, resulting in poor prognosis [17]. Here we found that IL-6 further promotes production of MMP-9 by ovarian cancer cells. These results are consistent with those recently generated from the studies of blockade of IL-6/STAT3 signaling pathway[17].

The current study is significant, because we have, for the first time, shown the molecular details for MMP-9 regulation by STAT3. Uno et al reported that the NF-κB-induced kinase (NIK) induced-mediated activation of non-canonical NF-κB signaling pathway (RelB/p52) plays a role in ovarian cancer progression through positively regulation of MMP-2 and MMP-9, resulting in enhanced NIK expression in ovarian cancer cells [18]. In this study, we showed that p-STAT3 directly interacts with a GAS element in *MMP-9* gene promoter to drive gene expression of MMP-9. Given a recent study showing that STAT3 can bridge RelB/p52 heterodimers and chromatin remodeling complex like histone acetylases, we speculate that p-STAT3 may also play a cooperative role in positive regulation of MMP-9 by assisting in recruitment of RelB/p52-containing transcriptional activator complex, as observed in placental cells [19, 30]. In other words, p-STAT3 could play multiple roles in regulation of MMPs in ovarian cancer cells. These results further suggest that use of SKOV3 cells may be extended to study epigenetic mechanisms involved in regulation of other MMPs such as MMP-2 [17]. MMP-2 breaks down type IV collagen and non-collagenous extracellular matrix proteins, such laminin, elastin, and fibronectin. Its increased expression is also associated with various pathological processes, including cancer invasion and metastasis [31]. Thus, it is highly likely that MMP-2 is regulated in a similar manner as MMP-9. As a result, a detailed molecular characterization of regulation of MMP-2 could further highlight STAT3 as an effective drug target for ovarian cancer. Moreover, our future directions could include testing effects of STAT3 inhibitors on proliferation and progression of ovarian cancer *in vivo*.

In conclusion, we report here that p-STAT3 directly interacts with *MMP-9* gene promoter to drive gene expression of MMP-9 cells. We propose that the strategy of modulating p-STAT3 activity may provide an effective therapeutic approach for treatment of ovarian cancer.
